# Angiofibroma of the spermatic cord: a case report and a review of the literature

**DOI:** 10.1186/1752-1947-5-423

**Published:** 2011-08-30

**Authors:** Panagiotis Dikaiakos, Adamantia Zizi-Sermpetzoglou, Spyros Rizos, Athanasios Marinis

**Affiliations:** 1 First Department of Surgery, Tzaneion General Hospital, 1 Zanni & Afentouli Street, 18536 Piraeus, Greece; 2 Department of Pathology, Tzaneion General Hospital, 1 Zanni & Afentouli Street, 18536 Piraeus, Greece

## Abstract

**Introduction:**

Cellular angiofibroma is a benign vascular neoplasm that typically arises in the paratesticular region in men and is easily confused with inguinal or scrotal hernia.

**Case presentation:**

We present a case of a cellular angiofibroma arising from the spermatic cord of a 74-year-old Caucasian man. Initially, the lesion was confused with a scrotal hernia, but imaging revealed a subcutaneous, inhomogeneous, but well-circumscribed lesion to the surrounding tissues with rich vasculature. Surgical resection of the lesion was performed. Histology revealed a benign tumor of vascular origin rich in fibroblasts.

**Conclusions:**

Angiofibroma can easily be confused with an inguinal hernia and should be differentiated from Schwann cell tumors, perineuromas, spindle-cell lipomas, aggressive angiomyxomas, angiomyofibroblastomas, solitary fibrous tumors, spindle-cell liposarcomas, and leiomyomas. A safe initial diagnosis is difficult because of its location, nature, and correlation with other structures of the area.

## Introduction

Cellular angiofibroma (AF) or angiomyofibroblastoma (AMF)-like tumor was first described by Nucci *et al*. in 1997 [1] and later, in 1998, by Laskin *et al*. [2] as a rare tumor distinguishable from AMF that occurs in the inguinal area, perineum, and scrotum in men and in the vulva in women. Although its origin is unknown, the suggested histogenesis is perivascular stem cells with a capacity for fatty and myofibroblastic differentiation [3]. Clinically, it can easily be mistaken for a sliding or scrotal hernia. The pathological and imaging features of AFs overlap those of AMF, solitary fibrous tumors, and angiomyxomas. We present a case of cellular AF of the spermatic cord and discuss the clinical, imaging, and histological findings as well as the differential diagnosis, with a brief review of the current literature on this topic.

### Case presentation

A 74-year-old Caucasian Greek man was referred to our surgical clinic for repair of a left inguinal hernia. The patient had noticed a gradually enlarging mass 10 years prior to presentation. His physical examination revealed an elastic, hard, slightly mobile mass that was initially confused with a scrotal hernia, although reduction maneuvers produced no result, even after the intramuscular administration of pethidine. No abnormal dermal findings were observed.

**Figure 1:**
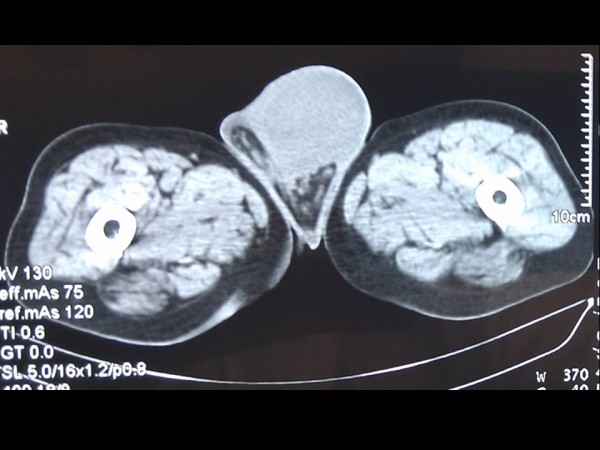
**Scrotal computed tomography demonstrating a mass in the left hemiscrotum**.

Ultrasonography showed the presence of a large (9 cm × 4 cm), rigid, inhomogeneous structure starting from the left inguinal space under the skin but not penetrating the corresponding hemiscrotum. Doppler sonography demonstrated prominent, rich vasculature. On computed tomography (CT), the lesion was observed to be round, with a diameter of 13 cm, inhomogeneous to the surrounding fat tissue of the anterior abdominal wall at the level of the left spermatic cord, and pushing away the left testis (Figure 1). No intravenous contrast medium was used because of allergy of the patient.

**Figure 2:**
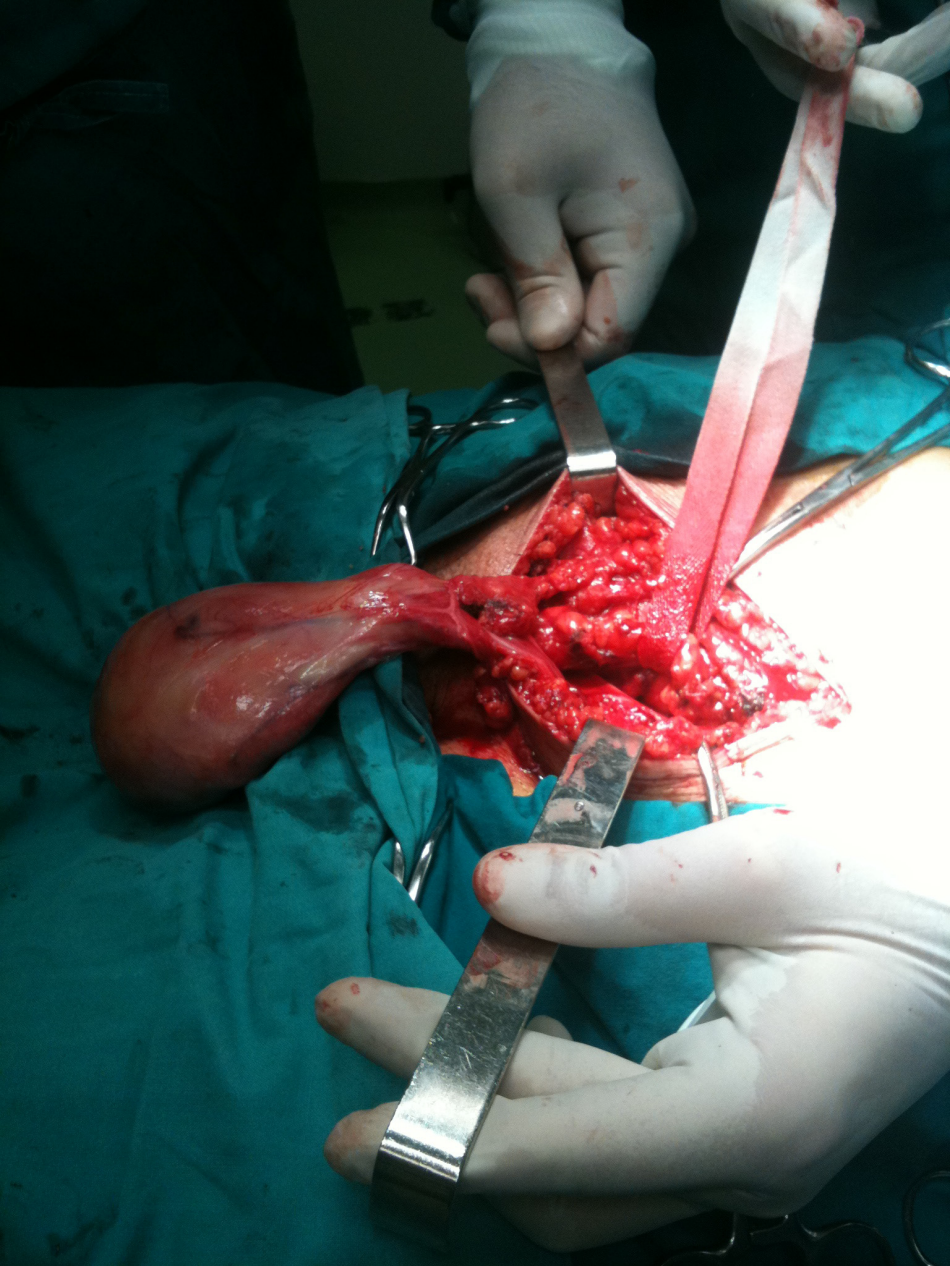
**Intra-operative photographs showing the relationship of the mass to the spermatic cord**.

Intra-operatively, the mass was found to be oval-shaped with dimensions 8 cm × 7 cm × 3 cm, well encapsulated, resembling fat tissue with rich vasculature, and it seemed to arise from the scrotal part of the spermatic cord without adherence to the ipsilateral testis (Figure 2). The mass was excised, and, because of the parallel presence of an inguinal hernia, typical mesh repair was performed.

**Figure 3:**
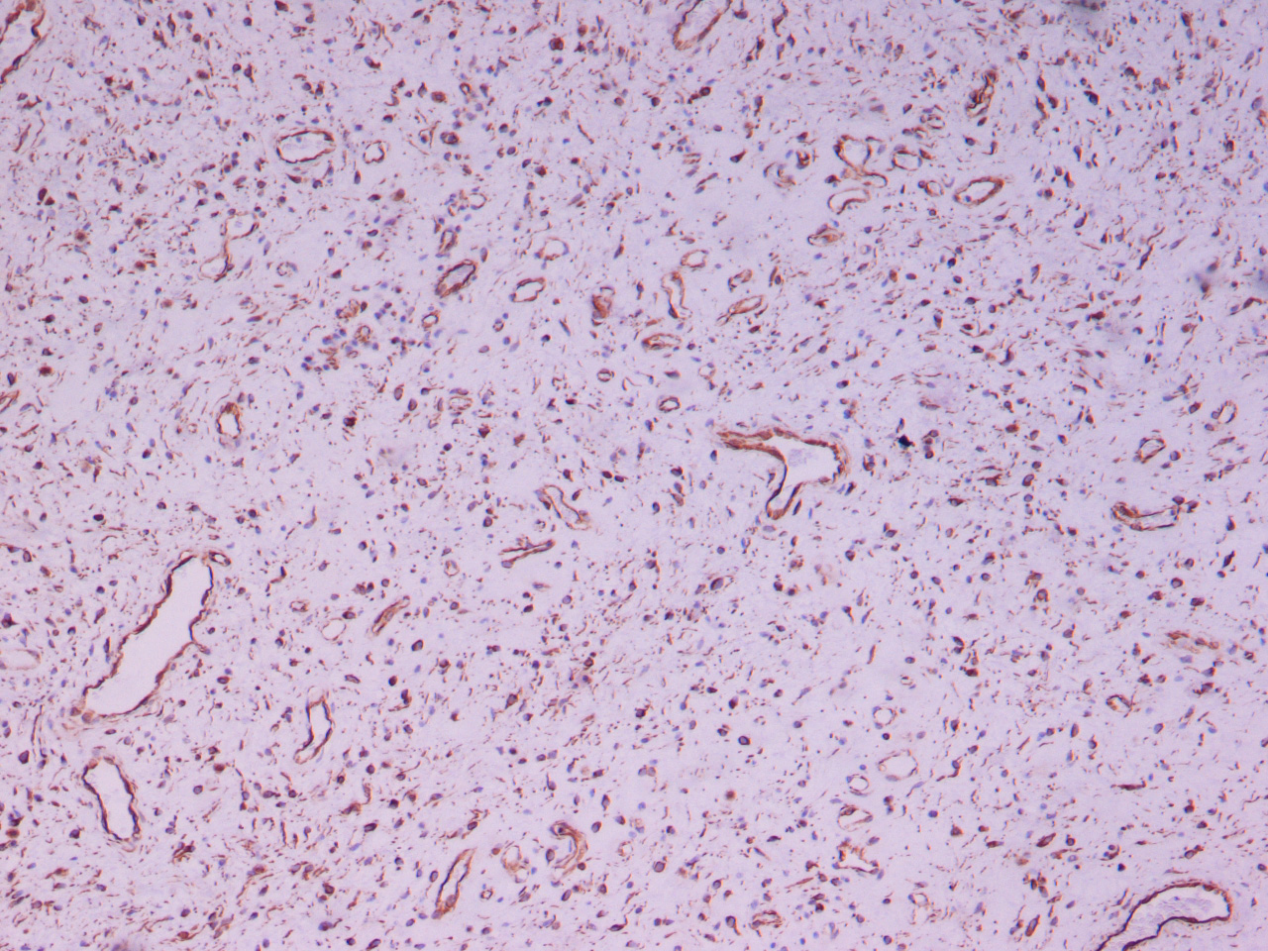
**Tumor cells show strong, diffuse expression of CD34 (hematoxylin and eosin stain; original magnification, ×20)**.

**Figure 4:**
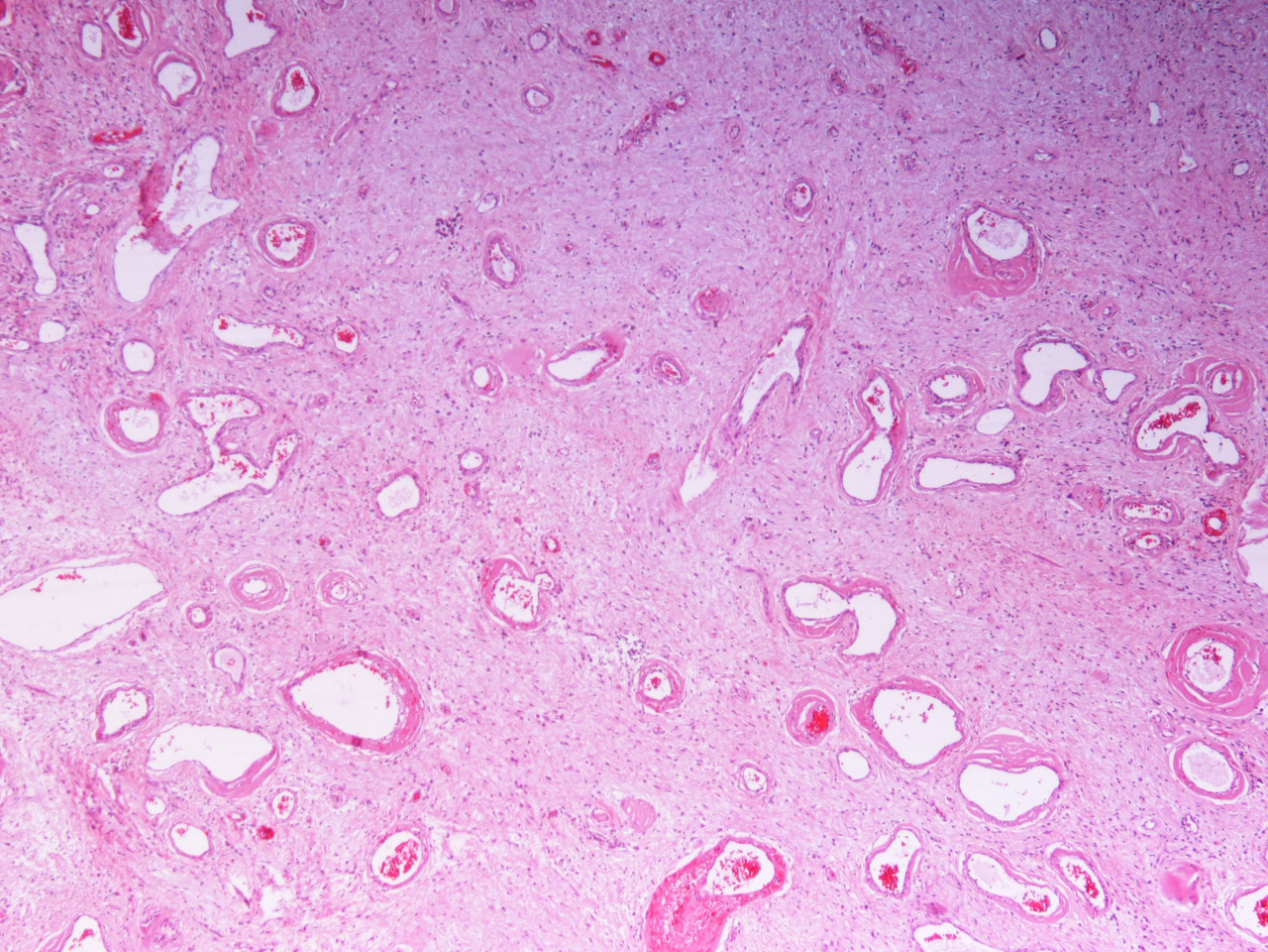
**Prominent dilated vessels with variably hyalinized walls and short spindle-cell fascicles (hematoxylin and eosin stain; original magnification, ×4)**.

**Figure 5:**
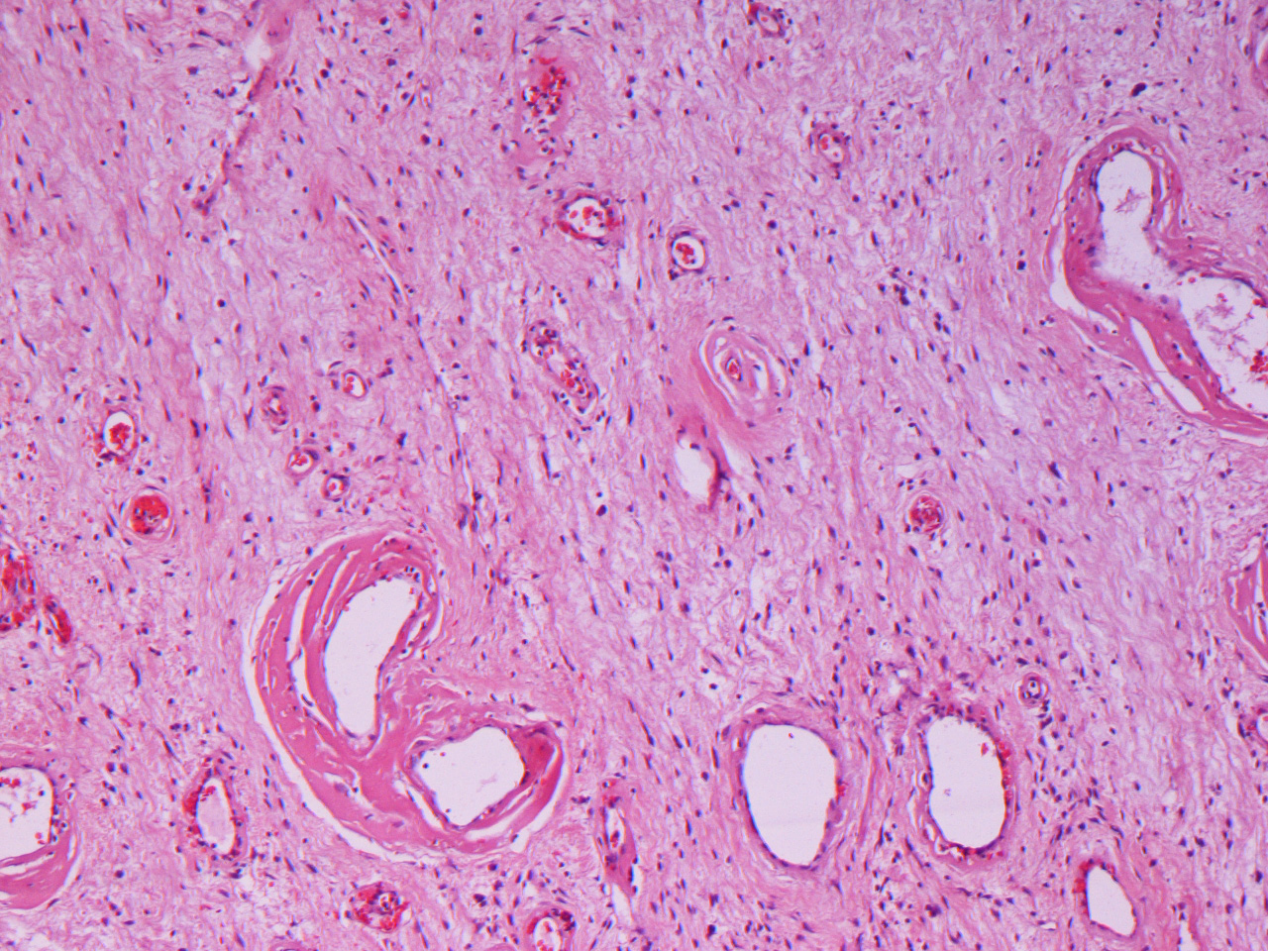
**Small and medium-sized vessels with hyaline walls (hematoxylin and eosin stain; original magnification, ×10)**.

Microscopically, the specimen consisted of loose fibrous tissue in which we found a large number of fibroblasts (vimentin- and CD34-positive and actin-, desmin-, and S100P-negative), inflammatory infiltration of lymphocytes, plasma cells, mast cells, and abundant capillaries, many of which with regenerating and degenerating forms (Figures 3 and 4). The walls of some tissues were thickened and those of others were hyalinized (Figure 5).

## Discussion

Embryologically, the mesoderm of the scrotum gives birth to various tissues; thus tumors arising from that area have high diversity, and confirming a safe diagnosis between a benign and malignant lesion is difficult. Cellular AF was first described in 1997 by Nucci *et al*. [1] as a distinctive, benign soft tumor of the vulva in women that is distinguishable from AMF. Later, in 1998, Laskin *et al*. [2] described the AMF-like tumor, namely, a mesenchymal tumor of the male genital tract resembling that described by Nucci *et al*. Finally, Iwasa and Fletcher [3] reported 51 cases of cellular AF occurring in both sexes and considered AMF-like tumors and cellular AFs to be similar entities. In that report, the patients' ages ranged from 22 to 78 years, with an average age of 53.5 years; the range of mass sizes was between 0.6 cm and 25 cm; and the primary location was in the subcutaneous tissue but was usually well marginated. The anatomic locations were most frequently the genital area (22 cases) in women and the inguinoscrotal area (19 cases) in men.

Histologically, the tumors are typically well circumscribed, quite cellular with spindle-shaped cells evenly distributed, and with short bundles of collagen. Less cellular areas are often associated with stromal edema or hyalinization, but significant pleomorphism and abnormal mitoses are absent. The numerous vessels observed are round, thick-walled, and hyalinized [3].

Immunohistochemical diagnostic procedures reveal that 60% of patients have slight expression of CD34 (vascular origin), 21% have spinal muscular atrophy (epithelial and/or glandular origin), and 8% reveal desmin (muscular origin) [3]. In our patient, the mass was an AF of vascular origin as revealed by its histopathological immunochemistry (vimentin- and CD34-positive and actin-, desmin-, and S100P-negative).

The diagnostic imaging workup includes a CT scan without specific findings for this entity [4], while on MRI scans AF may be hyperintense on the T2-weighted phase, depending on its origin and tissue composition (fat tissue, collagen, and spindle cells), or may show intense enhancement due to its rich vascularity [5].

It may be difficult to distinguish cellular AF from other tumors of the scrotum on the basis of radiological data only. The differential diagnosis includes tumors of Schwann cells, perineuromas, spindle cell lipomas [6], aggressive angiomyxomas (AAMs) [7], AMFs [8], solitary fibrous tumors (SFTs) [9], spindle-cell liposarcomas [10], and leiomyomas. Based on imaging, the differential diagnosis can be narrowed down to AAM, AMF, and SFT as follows: (1) AAM has a highly infiltrative pattern of growth, lower cellularity, and lower vascular growth and displays high signal intensity on T2-weighted MRI scans; (2) AMF exhibits high signal intensity on T2-weighted MRI scans but may appear slightly inhomogeneous, and the radiologic findings may be similar to those of cellular AF; and (3) SFT exhibits low signal intensity to isointensity for muscle tissue on T1-weighted MRI scans, intermediate to high signal intensity on T2-weighted MRI scans, and intense enhancement on gadolinium injection scans.

Surgical resection of the tumor is the therapeutic method of choice. Unfortunately, follow-up clinical data for cellular AF is limited, although recurrences have been reported [11]. A complementary resection must follow initial local excision if the tumor relapses.

## Conclusion

Cellular AF is a benign neoplasm of the scrotal and inguinal area, is rich in fibroblasts, and of vascular origin. A safe initial diagnosis is difficult because of its location, nature, and correlation with other structures of the area. It can easily be confused with a hernia, especially when the lesion slides toward the scrotum. Moreover, it is crucial to differentiate cellular AF from AAM and other spindle-cell neoplasms, since they exhibit malignant behavior with recurrences and metastases.

## Consent

Written informed consent was obtained from the patient for publication of this case report and any accompanying images. A copy of the written consent is available for review by the Editor-in-Chief of this journal.

## Competing interests

The authors declare that they have no competing interests.

## Authors' contributions

PD and AM analyzed and interpreted the patient data and drafted the manuscript. AZ performed the histological examination of the tumor. AM and SR critically revised the manuscript. All authors read and approved the final manuscript.
